# Hysteria: history of a conceptual and clinical pathomorphosis

**DOI:** 10.1192/j.eurpsy.2022.1397

**Published:** 2022-09-01

**Authors:** A. Sanz Giancola, C. Alvarez Garcia

**Affiliations:** Hospital Universitario Príncipe de Asturias, Psychiatry, Alcalá de Henares, Spain

**Keywords:** hysteria, history, pathomorphosis, transhistorical psychiatry

## Abstract

**Introduction:**

Transhistorical psychiatry defends that a psychic alteration can be interpreted as a cultural, historical and personal construction, subject to incessant variations.

**Objectives:**

A journey through the history of the disorder and the successive pathomorphoses it has undergone could provide us with a better understanding of it and explain the reason for the epidemiological trend towards a decrease in its diagnosis; and bring us closer to a universal definition of the phenomenon.

**Methods:**

Bibliographic review

**Results:**

The word hysteria and all its subsequent meanings, not only contain a particular conception of the pathology, but also reflect its different forms of presentation in specific periods of time. Hysteria is presented as a voluble material that can take on any form: from the wandering womb theory of classical Greece to the demonic possessions of the Middle Ages; from the neurological degeneration of Charcot (1825-1893) to the conversion and dissociation of Freud (1856-1939). With the entry of the 20th century, its dramatic clinic has been progressively overshadowed by somatoform disorders and emerging functional somatic syndromes. Today, it is practically unrecognisable and very difficult to diagnose, to the point of having disappeared as a term from the official classifications of our time.

**Conclusions:**

Hysteria is an entity that has not always been the same, neither in its conception nor in its manifestations. Depending on the socio-cultural context in which it is framed, it will be interpreted and expressed in different ways.

**Disclosure:**

No significant relationships.

